# Consultation Pricing of the Online Health Care Service in China: Hierarchical Linear Regression Approach

**DOI:** 10.2196/29170

**Published:** 2021-07-14

**Authors:** Ya-Ling Chiu, Jying-Nan Wang, Haiyan Yu, Yuan-Teng Hsu

**Affiliations:** 1 College of International Business Zhejiang Yuexiu University Zhejiang China; 2 Shaoxing Key Laboratory of Intelligent Monitoring and Prevention of Smart City Shaoxing China; 3 Research Institute for Modern Economics and Management Zhejiang Yuexiu University Zhejiang China; 4 College of International Finance and Trade Zhejiang Yuexiu University Zhejiang China; 5 Center for Health Decision Science Chongqing University of Posts and Telecommunications Chongqing China; 6 Research Center of Finance Shanghai Business School Shanghai China

**Keywords:** online health care industry, consulting pricing, reputation, wage level, hierarchical linear modeling, modeling, online consultation, pricing, linear regression, consultation, physician, eHealth

## Abstract

**Background:**

Online health care services are a possible solution to alleviate the lack of medical resources in rural areas, and further understanding of the related medical service pricing system would contribute to improvement of the online health care community (OHC). Although many studies have investigated the OHC, the impact of physicians’ reputations and wage levels on consulting prices in the OHC has rarely been discussed in the literature.

**Objective:**

This study was designed to explore the determinants of consulting prices in the OHC. We addressed the following questions: (1) Are the prices of online health consultation services affected by wage levels at the doctor’s location? (2) How does a physician’s online and offline reputation affect their consulting prices?

**Methods:**

Employing a large-scale sample of 16,008 doctors in China, we first used descriptive statistics to investigate the determinants of consulting prices in their entirety. Hierarchical linear modeling was then used to investigate the determinants of consulting prices in the OHC.

**Results:**

The empirical results led to the conclusion that if doctors have more elevated clinic titles, work in higher-level hospitals, have better online reputations, and/or have made more past sales, their consulting prices will be higher. Additionally, the wage level in the city in which the doctor is working determines their opportunity cost and therefore also affects consulting prices.

**Conclusions:**

The findings indicate that the characteristics of the doctor, the doctor’s online reputation, and past sales affect the consulting price. In particular, the wage level in the city affects the price of the consultation. These findings highlight that the OHC is important because it can indeed break through geographical restrictions and give rural residents the opportunity to obtain medical service from doctors in big cities. However, doctors from cities often charge higher fees because of their higher opportunity cost. The results reveal that one of the most important functions of the OHC is to reduce the medical disparity between urban and rural areas; however, planners appear to ignore the possibility that rural residents with lower incomes may not be able to afford such high medical consultation costs. Therefore, the government should consider providing incentives to encourage urban doctors to provide discounts to rural residents or directly offer appropriate subsidies.

## Introduction

### Background

Health care is a fertile field for research in the service area, and is an expensive, complex, and universally used service that substantially affects everyone [1]. Currently, certain activities related to health care are becoming more convenient due to ubiquitous internet connectivity; these new services may be referred to as online health care or telemedicine. Online health care may be defined as the use of medical information remotely via electronic communication to deliver health care services or improve a patient’s clinical condition. The online health care community (OHC) is growing quickly, which is changing the traditional channels and approaches to health care services [2]. The Telehealth Index: 2017 Consumer Survey indicated that 50 million Americans would prefer to switch their health care service provider to one that offered telemedicine, compared to 17 million in 2015. Additionally, approximately 60% of patients with chronic illnesses indicated that they would like to manage their conditions via remote diagnosis with their physicians regularly [3]. The popularity of the OHC in developed countries is unsurprising because it allows patients to save time and travel costs [4]. However, China’s overall per capita medical resources are relatively inadequate compared with those of more developed countries. According to the 2018 Organisation for Economic Co-operation and Development health resource data, China has only 1.80 doctors per 1000 people, which is relatively few compared with the numbers in the United States (2.60), the United Kingdom (2.80), Germany (4.30), and Norway (4.80) [5]. Thus, the development of the OHC is crucial not only for the sake of convenience but it is also an important means to reduce pressure on the entire medical system. Specifically, the OHC can reduce the length of hospital waiting times as well as rural-urban health disparities [6,7]. Therefore, it is worthwhile to devote attention to topics related to the OHC.

The OHC has developed very rapidly in China [8,9]. Up to 194.8 million people used online health care services in December 2016, accounting for 26.6% of the total number of internet users [10]. For these users, the three types of services with the highest usage rates were medical information inquiry (10.8%), online registration (10.4%), and online consultation services (6.4%). The first two services are usually available for free or for a small fee; in China, where the family doctor system is not as widespread as it is in developed countries, this has made online consulting services more important. However, online consulting services require payment of fees based on the doctor’s pricing. Additionally, online consulting prices are not regulated according to a certain pricing standard, and therefore differences in the prices charged by individual doctors can be very large. This raises an important question about whether the pricing is reasonable. Consumers want to be able to judge whether the consulting price is too high, while doctors want to set reasonable prices for consultation based on their personal conditions. In this study, we investigated the factors that might influence online health care consulting prices.

Two related points deserve further explanation. First, it is important to have a more complete understanding of the current status of China’s OHC to better understand the characteristics and limitations of this study. Xie et al [8] reported that there are 43 internet hospitals in China, 41 of which have licensed medical qualifications and 34 of which provide online consulting services. In addition to traditional hospitals adding various new remote services for patients, some internet companies have also obtained the necessary legal qualifications to provide medical services online. These internet companies have the advantage of being free from geographical and institutional restrictions. Specifically, they can hire qualified doctors from a variety of traditional hospitals across the country to provide medical services during their free time. In this study, data were collected from a well-known internet company represented by the Haodf website (*hao* means “good” and *dai fu* means “doctor” in Chinese). After appropriate data filtering (see “Data Collection and Sample Characteristics” section in the Methods for a detailed description), the total number of doctors in our sample was 16,008, from 1628 traditional hospitals in 30 provinces. With these data, we carried out a broad investigation of the determinants of consulting prices in China’s OHC.

Second, online health care consultation is substantially different from tangible products as it possesses certain characteristics of services, including intangibility, inseparability, variability, and perishability [11]. Although there has been substantial research on consulting fees [12-16], to the best of our knowledge, this study is the first to explore consulting fees in the OHC based on large-scale empirical data collection. Compared with traditional electronic market items, online health care consultation can be regarded as a typical credence good. When common products are purchased, customers can recognize their utility or quality after using them. In other words, people can distinguish whether the product is good or not. However, with credence goods, it is usually hard for customers to verify the quality of the product they have received [17-19]. For example, in the area of online health care consultation, when patients receive suggestions from the doctor, in general, it is hard for them to know for certain whether the doctor’s advice is appropriate for their personal situation. Similarly, it is difficult for patients to judge whether the pricing of consulting services is reasonable. This may induce the problem of moral hazard [20,21], which implies that physicians do not bear the full responsibility for the costs or risks associated with their providing online health care consultation and so may make less of an effort to reduce these costs. Although this study cannot propose a complete solution to the problem of moral hazard, it can provide several key factors that could help to explain physicians’ consulting prices. This would in turn help to reduce the occurrence of mispricing, either deliberately or through carelessness, which will ultimately contribute to further desirable developments in the OHC.

### Research Problem

The purpose of this study was to identify the determinants of online health care consulting prices. Owing to restrictions of data accessibility, two categories of determinants were included in our analysis. The first, relevant to economic factors, is the provincial wage level. The other category, obtained from the publicly available contents on the Haodf website, is related to the reputations of online and offline doctors. Reputation is a key indicator linked to trust [22], and is recognized as one of the primary factors affecting consumer behavior and seller performance, especially in online markets [23,24]. In the context of the OHC, a higher degree of information asymmetry may exist because OHC products have the characteristics of credence goods and items in online markets. Therefore, the particular nature of health care makes it necessary to consider both offline and online reputations so as to reduce information asymmetry. Previous empirical studies have focused on the correlation between reputation and price [15,23]. However, these studies investigated the online and offline reputations separately, thus not associating the combined offline reputation and online reputation with price. Given the multiple levels of data structure, in this study, hierarchical linear modeling (HLM) [25,26] was used to verify the relationships between online health care consulting prices and these determinants.

## Methods

### Research Model

#### Overview

**Figure 1:**
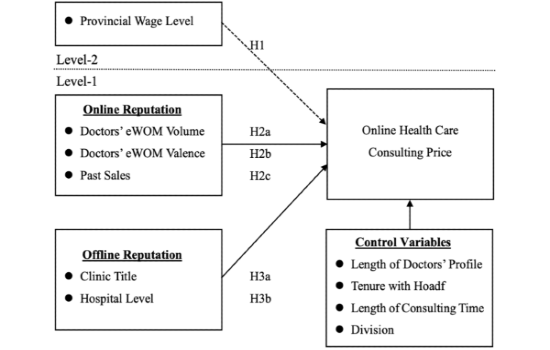
Proposed research model. eWOM: electronic word of mouth.

We developed an explanatory research model for this study, as shown in Figure 1. Several hypotheses related to online health care consulting prices are proposed. An integrated multilevel model of online health care consulting prices, economic factors, and online and offline reputation is proposed. The dotted line separates the Level-2 variable and the Level-1 variables. The arrows crossing the dashed line represent cross-level relationships with the dependent variable. We explain how the data were analyzed in this multilevel model in further detail below. Additionally, four control variables were included in this research model.

#### Theoretical Background: Service Pricing Approaches

Among the many approaches to service pricing [13,27,28], cost- and competition-oriented pricing strategies have dominated the pricing of services [29]. The cost-oriented pricing approach arrives at a service price by considering all of the costs and adding a desirable profit margin [29,30]. In the competition-oriented pricing approach, the price is set to meet that of the competitors or to accord with the market situation [31,32]. Although the simplistic nature of both approaches brings advantages of usability and rapidity, to accurately reflect the practices of complex business organizations, many extended approaches also take the characteristics of the service into consideration. For professional service providers such as physicians, Hoffman and Arnold [33] proposed an extended cost-oriented pricing approach, which includes not only the traditional factors of fixed costs, variable costs, and the profit desired but also a service characteristic premium. In particular, they take the service characteristics premium to be based on essentiality, durability, and tangible intrinsic value-added, which are also widely regarded as service characteristics. Similarly, Arnold et al [31] proposed a premium competition-oriented pricing approach in which the service price is equal to a differentiation premium plus the average competitor’s price. They suggest that the differentiation premium could be a function of availability, reputation testability, commitment incentive, and price sensitivity premiums. However, in practice, the precise definition of the differentiation premium should be dependent on the specific issue under consideration. Recently, studies have explored the factors that influence consulting pricing [14-16]. McLachlin [14] suggested that consulting engagements are successful if the client is satisfied that the consultant meets client expectations, which may enhance the consultant’s reputation and expectations of future revenue streams. These clients are more willing to pay a price premium for their services [34]. In addition, Momparler et al [15] showed that client satisfaction with the consulting team positively and strongly affects consulting fees. This satisfaction dimension may allow consultants to charge higher fees through the application of greater leverage while setting prices during the contracting process. Moreover, Lassala et al [16] performed a fuzzy-set qualitative comparative analysis to ascertain whether consulting-client satisfaction would explain differences in consulting fees and determine the conditions that lead to high consulting fees. Therefore, the aim of this study was to explore the critical determinants that affect online health care consulting prices, as this information can be used to improve the existing approaches to pricing. Specifically, these determinants can be regarded as components of the service characteristics premium or the differentiation premium in cost-oriented pricing or competition-oriented pricing, respectively.

We would like to further explain the importance of service characteristics for the analysis of online health care consulting prices by exploring differences between general services and those in the OHC. First, all service providers in the OHC are qualified doctors, and therefore this industry is professionally oriented; this suggests that service characteristics would be expected to play a critical role when providers price their services [33]. Second, almost all of the doctors in the OHC are online only part time, meaning that they provide online consultation during their nonworking hours. This service requires only a video or voice device and an internet connection; it is not necessary to rent an office or hire employees. Therefore, doctors have near-zero fixed costs, and their variable costs are closely related to their own opportunity costs. Third, all services are available online. Patients can find appropriate doctors according to information about specific doctors, without geographical restrictions. This implies that a doctor’s individual characteristics (eg, clinic title or online rating score) become more important for patients wishing to make the best decision in choosing a doctor [23,35]. In the following sections, we introduce the three types of determinants used in this study: economic factors, online reputation, and offline reputation. We then present the complete research model.

#### Economic Factors

We first discuss the effects of an economic factor: the provincial wage level. This is the local salary level at the doctor’s place of work. Intuitively, it seems obvious that a higher wage level usually means that doctors might experience higher opportunity costs when they provide online health care consulting services. As suggested by the cost-based approach [29,30], when doctors experience higher opportunity costs as a result of spending their time and effort offering a service, they will naturally request higher remuneration. Accordingly, we propose the following hypothesis:

Hypothesis 1: When the provincial wage level in the work area is higher, a doctor’s online health care consulting price will be higher.

#### Online Reputation Factors

Given that there are fewer repeated interactions between two transacting parties on the internet, Ba and Pavlou [23] pointed out that building appropriate feedback mechanisms can bring about a calculus-based credibility trust. There are three sources of trust in the business world: familiarity, calculativeness, and values [36]. In addition, two types of trust can be distinguished: benevolence and credibility [22,37]. In the context of an online transaction, Ba and Pavlou [23] suggested the term “calculus-based credibility trust” to express the relevant characteristics of this kind of market. Moreover, information asymmetry on the internet generally induces transaction-specific risks, but trust can mitigate these risks and thereby generate price premiums for reputable sellers. However, a major limitation in obtaining a sample from secondary data rather than using an experimental design is that it is not easy to measure trust perceptions with the former approach; therefore, some research such as the second study in Ba and Pavlou [23] has focused on evaluating the existence of a direct relationship between feedback mechanisms and price premiums. It is worthwhile to examine the meanings of the notions of feedback mechanisms and price premiums more carefully. First, in our context, to make it easier to mitigate the same problem of information asymmetry as can be found with other internet platforms, the Haodf website has provided a feedback mechanism that can produce reputational outcomes resulting from each agent’s behavior. Second, following Ba and Pavlou [23], the price premium of one specific product is defined as the amount of money above the average price charged by different sellers. Because this study only considers one type of product, it is not necessary to subtract the average price to effectively compare the products at different price levels. Exploring the variation in consulting prices is essentially the same as examining the providers’ price premiums. Therefore, higher consulting prices may be viewed as compensation to doctors for creating a good reputation, whereas lower consulting prices may be viewed as compensation to patients for bearing a greater transaction risk. As elaborated above, we believe that if the doctor has a better online reputation, their online consulting price will be higher. In the context of the OHC, the doctor’s online reputation is based on patients’ overall assessment of the doctor and indirectly reflects their level of trust in the doctor [23,38]. We will introduce three variables related to doctors’ online reputations; the values of these variables can be determined based on information collected from the Haodf website.

The online reputation reflects a doctor’s past actions, and the site allows patients to directly evaluate doctors and share their assessments with others. In general, this type of online reputation can be measured through assessing a doctor’s electronic word of mouth (eWOM) [39]. Two major attributes of eWOM, volume and valence [40,41], are considered in this study. A large volume of reviews makes a doctor stand out from the crowd, which might attract more attention from patients [40,41], and the valence (a positive or negative direction) of the patient reviews usually represents the quality of the doctor reviewed [42-44]. Moreover, an effective feedback mechanism, which can help buyers distinguish between sellers, can create price premiums [45]. In the buyer-and-seller relationship literature, Ba and Pavlou [23] found that there is a positive relationship between a seller’s reputation and the price asked on eBay. This suggests that a better eWOM will come with a more favorable evaluation of a doctor and make patients more likely to pay higher prices. In addition, a doctor’s past online sales partially reflect popularity, which is a signal of consulting quality [46-48]. Classical observational learning studies have found that customers tend to take cues from selections made by their peers because they infer the quality of products from such peer selection [49,50]. A higher past sales volume can also increase patients’ trust and facilitate online transactions by reducing perceived risk. In this way, we expect that the past sales volume will positively affect the price of online health care consultations. Therefore, we propose the following hypotheses:

Hypothesis 2a: When a doctor has a larger eWOM volume, their online health care consulting price will be higher.

Hypothesis 2b: When a doctor has a more favorable eWOM valence, their online health care consulting price will be higher.

Hypothesis 2c: When the volume of a doctor’s past online sales is larger, their online health care consulting price will be higher.

#### Offline Reputation Factors

Reputation is one of the important service characteristics [31]. A physician’s offline reputation is a useful signal that can increase patient trust. For example, one patient’s positive experience can increase trust in the doctor by other patients and minimize their perception of risk [51]. In the OHC context, a physician’s offline reputation includes two aspects: (1) clinic title, which is the medical capability of the doctor assessed by the government agency based on the doctor’s comprehensive ability; and (2) the level of the hospital with which the doctor is affiliated, which also reflects the doctor’s ability. The clinic title is nationally standardized with four levels: resident physician, attending physician, associate physician, and chief physician (listed here from the most junior to the most senior level). Similarly, there are corresponding levels of the hospitals that doctors belong to: Level III, Level II, and Level I [52]. Level III is the highest level; hospitals at this level have more beds, better equipment, highly skilled doctors, and more comprehensive health care quality [53,54]. A doctor with a higher clinic title or coming from a high-level hospital usually translates to more experience, which leads to increased trust in the doctor, a situation that should reduce information asymmetry risks and result in a price premium for providers. Thus, we propose the following hypotheses:

Hypothesis 3a: When a doctor has a higher-level clinic title, their online health care consulting price will be higher.

Hypothesis 3b: When a doctor is from a higher-level hospital, their online health care consulting price will be higher.

#### Control Factors

Four control variables were included in this research model. First, we considered the length of the personal profile as benefits accrue to the patient if the information can be obtained without additional search costs. In general, if there are more words in the profile, then more details about the doctor, such as academic qualifications, clinical experience, area of expertise, or other capabilities, will be accessible. The added depth of information can increase a patient’s confidence in making the decision to use the doctor’s services [55]. Second, since the Haodf website has been established for more than 10 years, the length of time the doctor has had their profile activated on the website was also considered in our model. We refer to this variable as tenure with Haodf; tenure is generally used in studies related to the OHC [7,53,56]. Third, due to the fact that the length of time that is considered to constitute a consultation varies (eg, some doctors charge per 15 minutes and some charge per 10 minutes), we added the length of consulting time as a control variable to reduce its influence on our analysis. Finally, we also took the classification of the doctor’s expertise into consideration and therefore added the doctor’s division as another control variable. Given the complexity in assessing a doctor’s expertise, we used the classification method provided by the Haodf website to divide the doctors into 10 divisions. Controlling this categorical variable was expected to reduce the effects of different types of expertise on online health care consulting prices.

### Data Collection and Sample Characteristics

We collected a large amount of public data from the Haodf website [57] to explore our research questions. Founded in 2006, the Haodf website is the largest doctor-patient interaction platform in China and is considered to be the most professional and trustworthy OHC. More than 490,000 physicians from 7500 hospitals nationwide are included on this website, with 145,000 physicians having completed real-name registration on the website as of 2017. In addition, we chose this OHC for two reasons. First, online doctor consultation services on Haodf have involved charges since the middle of 2016. Through the Haodf website, patients can consult with their doctor via text, voice, or video as needed regardless of time and geographical restrictions. These services are offered to patients for different fees. Since telephone consultation services were the most widely used mode during the data collection period, this study mainly selected the telephone consulting price for discussion. We can also observe the telephone consulting price per doctor directly on the website. Second, since Haodf is one of the most popular OHCs in China, many recent studies have used data sources from this website to explore various research questions [7,52,53,56,58,59], which means that the data collected from this site are reliable and representative. Our data collection procedure is described in further detail below.

**Figure 2:**
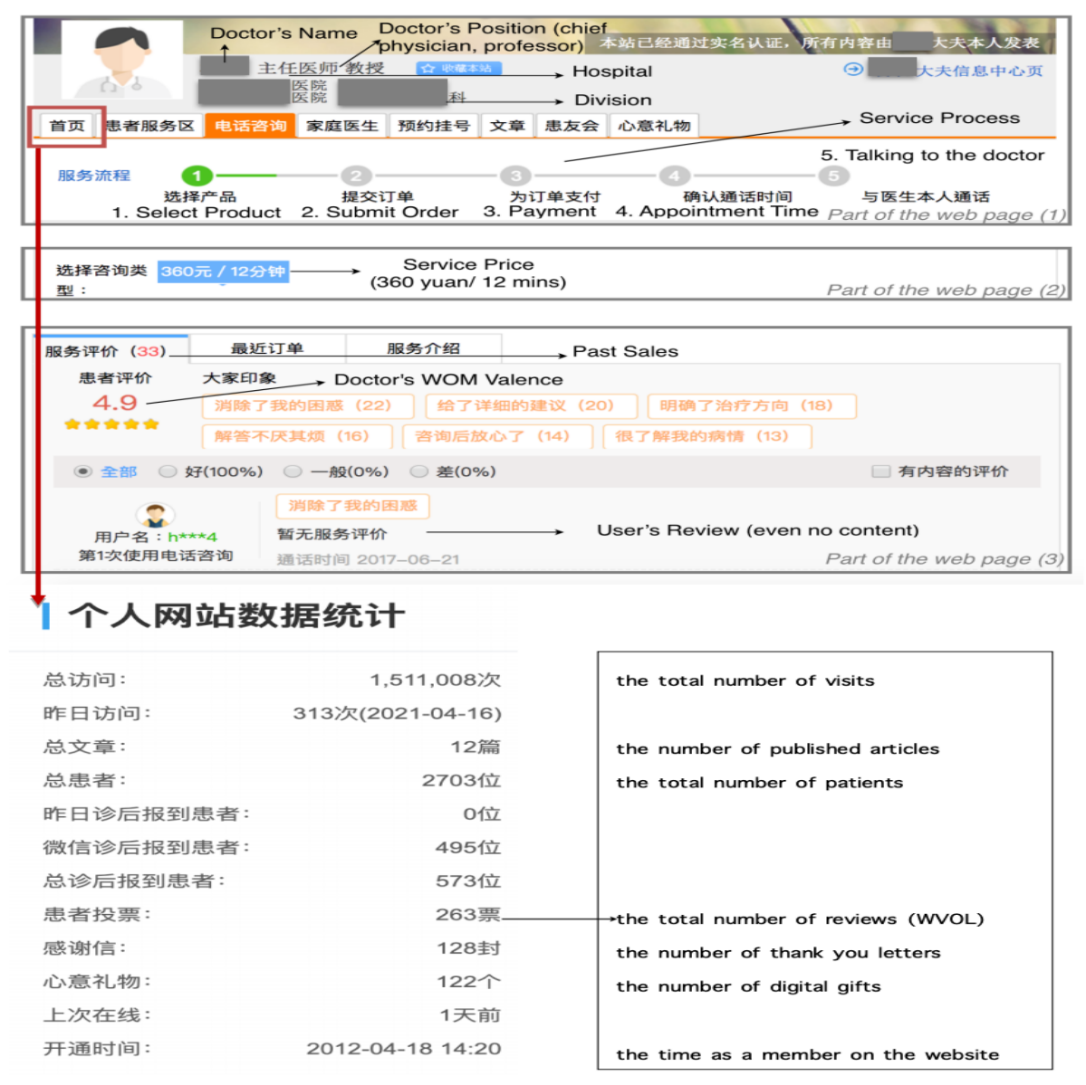
Haodf website screenshot. WOM: word of mouth; WVOL: volume of electronic word of mouth.

We adopted web crawler technology for the purpose of exploring more than 423,000 doctors’ profiles on the Haodf website [57]. Within the sample, not every doctor provided online consulting services. Specifically, only 44,780 of the 423,000 doctors offered this kind of service and announced the related prices on their personal pages. The website is designed such that when a patient pays for a service, one review record will be added to the doctor’s page, even if the patient does not comment. Thus, we can observe the number of past sales through the number of reviews on each doctor’s page. To ensure that real tradable prices were used in the analysis, the final sample considered only 16,008 doctors who had actually provided at least one online consulting service to a patient. Data from the Haodf website have been used in many recent studies [53,56]; therefore, readers interested in obtaining more information on the Haodf website can refer to this literature. Additionally, for convenience, we also provide screenshots relevant to this kind of service on the Haodf website in Figure 2 to illustrate the form in which the doctor’s basic information (eg, name, position, and division), details about the service process, the consulting price, and patients’ reviews can be found.

**Figure 3:**
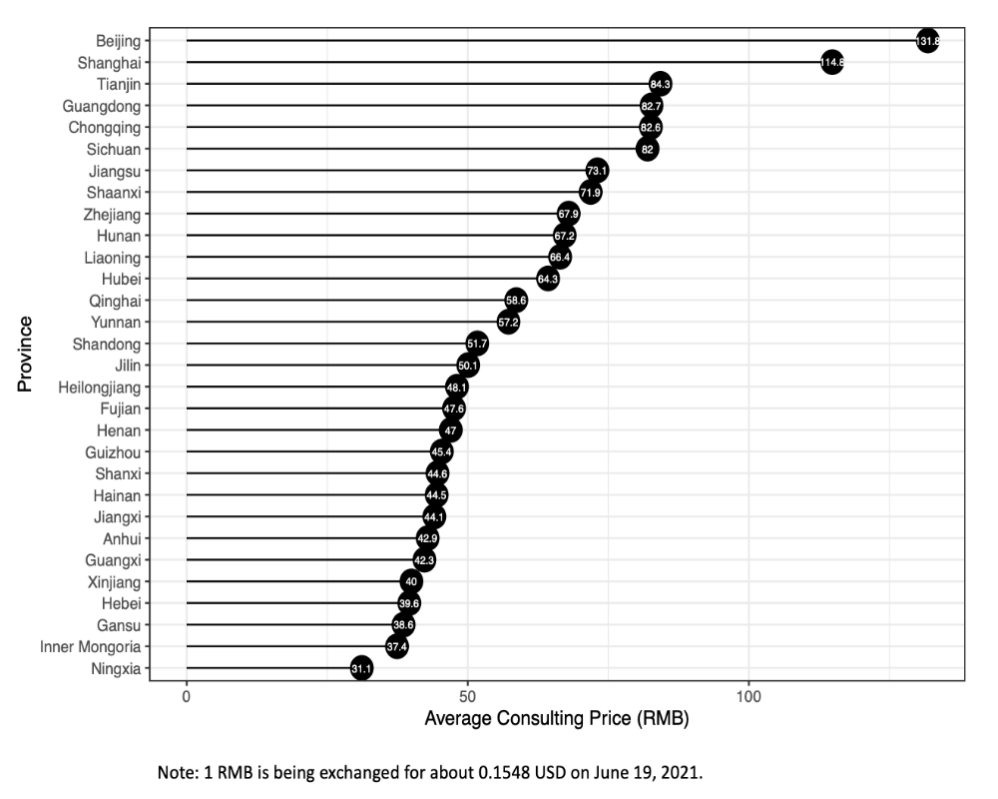
Online health care consulting prices in different provinces.

Certain characteristics of the sample deserve further mention. First, the total past sales, or the number of patients who had paid for a consulting service on the Haodf website, was 320,265. In other words, many people had used this type of service, which also reflects the degree to which the OHC has been accepted in China. Second, these 16,008 doctors came from 1628 different hospitals, representing 10 different kinds of professional divisions, and were from 30 different provinces in China. This shows that our sample was not confined to a particular organization, medical specialty, or geographical area. Third, this online health care consulting service charged on average 84.7 yuan (about US $13; 1 yuan=~US $0.15) per consultation, or 7.59 yuan per minute. Notably, the consulting price was clearly affected by the province in which the doctor’s hospital was located, as shown in Figure 3. For example, the average consulting price in Beijing was nearly double that in more than half of the other provinces. To take this difference into account, the provincial wage level was included in our model to make the differences in online consulting prices in the various geographical areas more apparent.

### Measures

#### Dependent Variable: Consulting Price

The dependent variable in this study was the telephone consulting price, which is the price plan set by doctors who provide online health care telephone consulting services. For example, a doctor might charge 200 yuan per 10 minutes or 500 yuan per 15 minutes. The patient can then make an appointment with the doctor directly and pay the relevant fees online. It is important to note that the patient always pays for a block of time instead of paying for the precise amount of time spent in consultation. In other words, when the patient chooses a price plan such as 500 yuan per 10 minutes, they will be charged 500 yuan even if the actual consulting time is less than 10 minutes. To guarantee the fairness of the transaction, the Haodf website acts as a third-party impartial institution. At the appointed time, the patient makes a call to the Haodf website and an employee assists them in contacting the doctor for the telephone consultation. After completing the telephone consultation, the patient writes a comment about the service that has been provided. If the patient does not write a review, the system generates a record with an empty comment. In light of these facts, we defined the telephone consulting price as the listed price (in yuan) per instance of the online health care consulting service provided by a doctor, and the listed length of consulting time was added to the model as a control variable. In these terms, among the 16,008 doctors, approximately 70% charged a price less than 100 yuan, but 116 doctors charged an amount more than or equal to 500 yuan. In addition, the mean price was 84.7 yuan, but the median was only 57.5 yuan. These two results indicate that the distribution of online health care consulting prices has a significantly positive skewness; therefore, we used the online health care telephone consulting price in the natural logarithmic form in our analysis model.

#### Independent Variables

##### Wage Level

**Figure 4:**
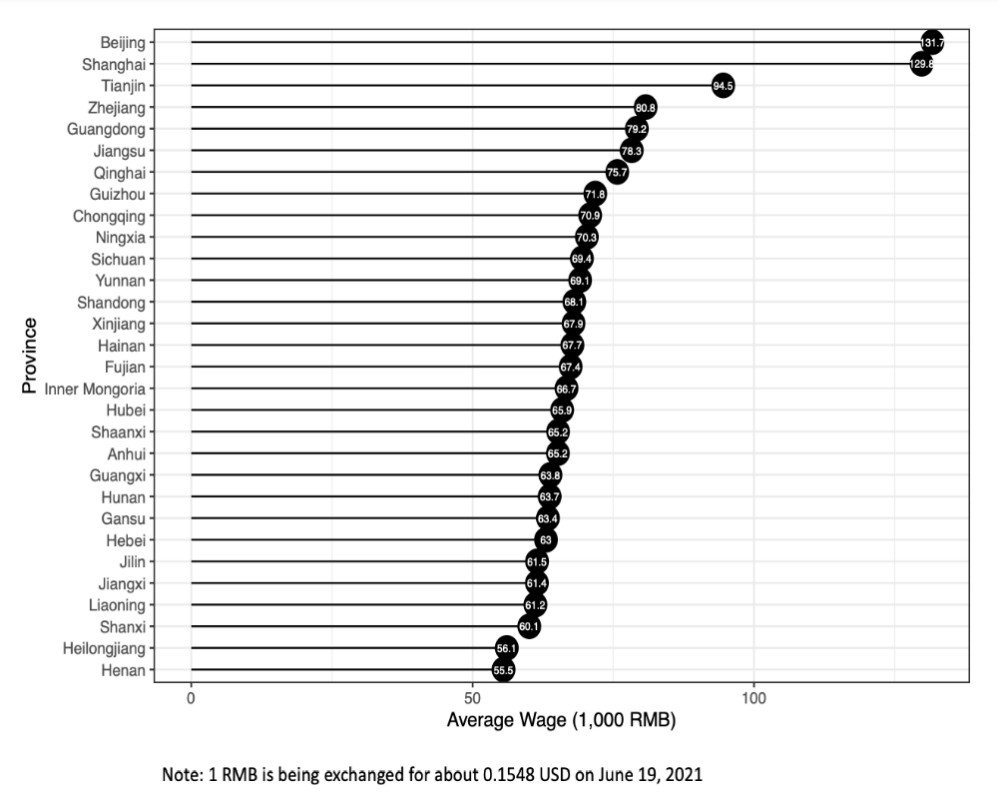
Wage levels in different provinces.

The doctor’s wage level was measured using the provincial average currency wage for employees in 2017. All doctors from the same province in the sample were related to the same provincial wage level. Therefore, the provincial wage level was regarded as a level-2 or group-level variable. We obtained the provincial wage level from the Wind Economic Database [60] and formally used the variable WAGE for representation. Figure 4 shows the wage levels in 30 provinces; these figures also indicate the significant relationship between the telephone consulting prices and the provincial wage level. For example, Beijing, Shanghai, and Tianjin are not only the top three cities in terms of average telephone consulting prices but are also the top three in terms of the provincial wage level.

Besides the level-2 variable, our model further included five level-1 or individual-level variables: eWOM volume, eWOM valence, past sales, clinic title, and academic rank.

##### eWOM Volume

The doctor’s online reputation was measured using the volume of eWOM (WVOL) calculated by the number of reviews of the doctor. Because the distribution of the variable WVOL had significantly positive skewness, we used the natural logarithmic in our analysis model.

##### eWOM Valence

The doctor’s online reputation was used to measure their overall score on the website. Specifically, after a patient receives a doctor’s service, they can indicate that the doctor has a certain professional ability to evaluate the doctor, giving the doctor a score of 1 to 5 (5 being the best). The eWOM valence (WVAL) was then measured as the mean of the overall ratings of doctors.

##### Past Sales

The doctor’s online reputation was also used to measure the past sales of their telephone consultation services. Even if the review content is empty, the system keeps all records of online health care consulting reviews created when users have finished receiving a service. Hence, based on the number of these reviews, we defined the variable PSALE as the number of patients who paid for the online health care services provided by doctors.

##### Clinic Title

The doctor’s offline reputation was measured using the doctor’s clinic title, which is the medical ranking of the doctor as evaluated by the government according to the doctor’s comprehensive abilities. In China, the doctor’s clinic title can be specified as chief physician, associate chief physician, attending physician, or resident physician. We defined the clinic title as a dummy variable (CL), in which the positions of chief physician or associate chief physician are coded 1 and others are coded zero [7].

##### Hospital Level

The doctor’s offline reputation was measured by the level of hospital to which they belonged. In this study, we defined hospital level as a dummy variable (HL), with 1 assigned to a doctor from a tertiary hospital and 0 otherwise [7].

#### Control Variables

We employed four control variables in this study. First, doctors can provide their personal profile information such as education, experience, and expertise for public reference on the Haodf website. We calculated the number of words in each profile to represent the level of individual information disclosure, which represented the value for the variable WORD. Second, because the Haodf website had been established for more than 10 years, how long the doctor had been a member might have affected their behavior on the website. To control for time effects, the TENURE variable was defined as the tenure with the Haodf website [56], which was measured as the number of days the doctor had their own profile activated on the website. Third, because the amount of time considered to constitute a single consultation was different for different doctors, it was necessary to take this difference into consideration. Therefore, LCT was defined as the length of a single consulting time. Finally, doctors have different major specialty areas such as surgery, internal medicine, pediatrics, and Chinese medicine. To reduce the effect of the characteristics of the doctor’s specialty, we further added another control variable, DIV, which is a categorical variable with 10 major divisions, based on information reported on the website. The detailed variable definitions and measurements are shown in Table 1. It is important to note that the distributions of several variables were positively skewed; for example, approximately 80% of doctors had fewer than 500 words in their profile, but nearly 5% of doctors had more than 1000 words. To alleviate the problem of data being positively skewed in our analysis, the variable was transformed to the natural logarithm form and the letter “L” was added at the beginning of its code (eg, LWORD is the variable WORD in the natural logarithmic form).

**Table 1:** Variable measurements and sources.

Code	Variable	Measurements	Source
PRICE	Consulting price	The listed price of the online health care consulting service provided by doctors	Haodf
WAGE	Provincial wage level	The provincial average currency wage of employees in 2017	Wind Economic Database [60]
WVOL	Doctor’s eWOM^a^ volume	The number of reviews provided by patients indicating the doctor’s professional ability on the website	Haodf
WVAL	Doctor’s eWOM valence	Mean of the overall ratings in patients’ reviews of the doctor	Haodf
PSALE	Past online sales	Number of patients who had paid for the doctor’s online health care service	Haodf
CL	Clinic title	Clinic title of the doctor, dummy variable: CL=1 if the doctor’s position was chief physician or associate chief physician; 0 otherwise	Haodf
HL	Hospital level	The level of hospital to which the doctor was affiliated, dummy variable: HL=1 if the doctor was from a tertiary hospital; 0 otherwise	Haodf
WORD	Length of doctor’s profile	Number of words in the doctor’s personal profile	Haodf
TENURE	Tenure with Haodf	The doctor’s tenure with the Haodf website (in days), calculated based on the data download date minus the doctor’s registration date on the website	Haodf
LCT	Length of consulting time	The listed length of one occasion of the consulting service (in minutes) provided by doctors	Haodf
DIV	Division	The doctor’s division as categorized by the website, including internal medicine, surgery, gynecology/obstetrics, pediatrics, orthopedics, ophthalmology, oral health, cancer, Chinese medicine, and others	Haodf

^a^eWOM: electronic word of mouth.

### Statistical Analysis: HLM

The hierarchical level of grouped data is a common consideration in many research contexts [61]. For example, to explore how job satisfaction influences work performance, different employees’ individual characteristics are usually taken into consideration. In addition, since workers are part of different teams, the cohesiveness of each team might also be an important factor in the model. However, using simple linear regression techniques to perform such analyses may be insufficient given the lack of consideration to shared variance. To overcome this problem, HLM is one of the most popular methods that can simultaneously handle the relationships among variables both at the within-group and between-group levels [25,26]. In this study, the sample involved a hierarchy with two levels. Specifically, the higher level of the hierarchy (level 2) includes the provincial wage, and a lower level of the hierarchy (level 1) includes other variables (eg, consulting price, clinic title, or academic rank). All level-1 variables are nested within level-2 groups and have a share in the common impact of the level-2 variable. We therefore performed HLM as our statistical analysis, which explicitly takes into account this cross-level data structure. We implemented the related HLM analyses through the multilevel and nlme packages in R.

To examine whether our data met the prerequisites for HLM analysis, we adapted a one-way analysis to identify the within-group and between-group variance in the dependent variable. Formally, the relevant null model is as follows:

Level 1: LPRICE_ij_=β_0j_+r_ij_**(1)**

Level 2: β_0j_=γ_00_+u_0j_

where *LPRICE_ij_* is the natural logarithm form of the consulting price measured for doctor *i* in province *j*, and *β_0j_* is the mean of *LPRICE* for province *j*. In the combined form, the model is *LPRICE_ij_*=*γ_00_*+*u_0j_*+*r_ij._* In this way, the null model essentially means that the dependent variable is a function of a common intercept *γ_00_* and there are two error terms: the between-groups error term *u_0j_* and the within-group error term *r_ij_*. In addition, we denote the variances *r_ij_* and *u_0j_* as *τ_00_* and *σ^2^*, respectively. Based on the general assumption of HLM, *cov*(*r_ij_*, *u_0j_*)=0, the variance of LPRICE can be partitioned into between-group variance (*τ_00_*) and within-group variance (*σ^2^*). We can then calculate the intraclass correlation coefficient (ICC), which is the ratio of between-group variance to total variance, as follows:

ICC=τ_00_/τ_00_+σ^2^**(2)**

Once significant between-group variance in the dependent variable was determined, two types of HLMs were used to test the hypotheses of this study. First, we considered the random intercept model as follows:



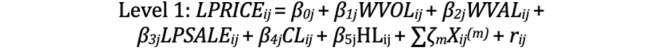

Level 2: β_0j_=γ_00_+γ_01_LWAGE_j_+u_0j_

β_kj_=γ_k0_, k=1, 2,…,5 **(3)**

where *LPRICE_ij_* is the natural logarithm form of the consulting price measured for doctor *i* in province *j*, and *β_0j_* is the mean of LPRICE for province *j*. In addition, LWVOL, WVAL, LPSALE, CL, and HL are independent variables, and *X_ij_^(m)^* represents the control variables, including LWORD, LTENURE, LCT, and DIV. The relevant definitions of the variables are shown in Table 1. We added the letter “L” at the beginning of the variable code to identify a variable in natural logarithm form. To further clarify the meaning of the random intercept model, we also rewrote it as the combined form:



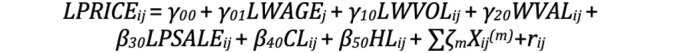



where *γ_00_, u_oj_,* and *r_ij_ represent* a common intercept, the between-groups error term, and the within-group error term, respectively. Compared to the traditional ordinary least-squares regression model, the random intercept model has additional between-groups error terms, which allows the intercepts to be different among groups. However, the impact of independent variables on the dependent variable is fixed. In other words, for one specific independent variable, the coefficients or slopes (*β_kj_*) in all groups (provinces) are the same. The second type of HLM, described below, will relax the fixed effect of *β_kj_*.

The second HLM used in our analysis was the random coefficient model. The setup of level 1 in the random coefficient model is the same as that in the random intercept model, but at level 2 it involves an extra error term in *β_kj_*. This model can be written as follows:



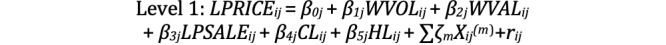

Level 2: β_0j_=γ_00_+γ_01_LWAGE_j_+u_0j_

β_kj_=γ_k0_+u_kj_, k=1, 2,…,5 **(4)**

where *u_kj_* is the error term and other symbols have the same definitions as in the random intercept model. The combined form of the random coefficient model is:



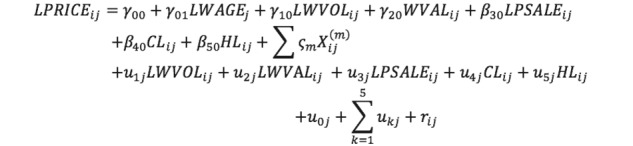



Therefore, the random coefficient model has a more complex structure of error terms compared to the random intercept model, which allows not only the intercepts but also the coefficients of each independent variable to be different among various groups. Both the random intercept and random coefficient models were used in our empirical study.

## Results

### Descriptive Statistics

The total number of samples used in this study was 16,008, and the relevant descriptive statistics (means, SDs, minima, maxima) for the sample are shown in Table 2. Note that natural logarithm transformation was used to mitigate the issue of skewed distribution. In the following discussions of the HLM, several variables, including PRICE, WAGE, WVOL, PSALE, WORD, and TENURE, will be presented on the transformed scale, and others will be presented on the original scale. In addition, since CL and HL are dummy variables, their means represent the proportion of variable values equal to 1 for the entire sample. Overall, 72.3% of the doctors in our sample were chief physicians or associate chief physicians, and 90.2% of the doctors were from tertiary hospitals.

**Table 2:** Descriptive statistics of variables (N=16,008).

Variable	Mean (SD)	Minimum	Maximum
Consulting price (PRICE)	84.699 (82.257)	1	1500
Provincial wage level (WAGE)	90,961.600 (29,497.110)	55,495	131,700
Doctor’s eWOM^a^ volume (WVOL)	62.884 (104.995)	0	1,746
Doctor’s eWOM valence (WVAL)	3.997 (0.349)	2.8	5
Past online sales (PSALE)	20.007 (98.232)	1	4062
Clinic title (CL)	0.723 (0.448)	0	1
Hospital level (HL)	0.902 (0.298)	0	1
Length of doctor’s profile (WORD)	381.407 (696.944)	41	27,301
Tenure with Haodf (TENURE)	1780.000 (988.839)	12	3402
Length of consulting time (LCT)	11.121 (2.763)	5	30

^a^eWOM: electronic word of mouth.

The correlations between major variables are listed in Table 3. All correlation coefficients were significantly positive at the 0.1% level, except for the correlation coefficient between the doctor’s hospital level and the length of the doctor’s profile, which was not significant.

We first verified whether the hypothesized determinants of the online health care consulting price are truly influential. The doctors were divided into two groups based on the median for the four continuous variables or the category for the two dummy variables. We then determined whether the average price of the two groups was the same. For example, since the median of WVOL was 27, the full sample was divided into two groups: those with higher WVOL and those with lower WVOL. The average PRICE for the higher group was 109.9, whereas that for the lower group was only 59.6. Table 4 shows the relevant results for all determinants. Moreover, we also adopted two-sample *t* tests to verify the significance of the difference of means. In each case, the means of the two groups were not equal at the 0.1% significance level. Therefore, these preliminary findings revealed that a doctor with a higher offline reputation (whether in terms of clinic title or hospital level), receiving a greater eWOM volume, a higher eWOM rating, having made more sales in the past, or coming from a higher-wage province generally leads to their claiming a higher online health care consulting price. In the following sections, we provide more empirical evidence to support the argument that these determinants significantly affect the online health care consulting price, and how they affect the price.

**Table 3:** Correlation analysis

Variable			LPRICE^a^		LWAGE^b^		LWVOL^c^		WVAL^d^		LPSALE^e^		CL^f^		HL^g^		LWORD^h^		LTENURE^i^		LCT^j^
**LPRICE**																					
	*r*	1.000		0.388		0.461		0.429		0.489		0.359		0.202		0.353		0.286		0.390	
	*P* value	—^k^		<.001		<.001		<.001		<.001		<.001		<.001		<.001		<.001		<.001	
**LWAGE**																					
	*r*	0.388		1.000		0.163		0.162		0.290		0.069		0.038		0.147		0.166		0.151	
	*P* value	<.001		—		<.001		<.001		<.001		<.001		<.001		<.001		<.001		<.001	
**LWVOL**																					
	*r*	0.461		0.163		1.000		0.722		0.588		0.252		0.123		0.330		0.320		0.138	
	*P* value	<.001		<.001		—		<.001		<.001		<.001		<.001		<.001		<.001		<.001	
**WVAL**																					
	*r*	0.429		0.162		0.722		1.000		0.419		0.180		0.230		0.258		0.134		0.100	
	*P* value	<.001		<.001		<.001		—		<.001		<.001		<.001		<.001		<.001		<.001	
**LPSALE**																					
	*r*	0.489		0.290		0.558		0.419		1.000		0.195		0.092		0.254		0.350		0.252	
	*P* value	<.001		<.001		<.001		<.001		—		<.001		<.001		<.001		<.001		<.001	
**CL**																					
	r	0.359		0.069		0.252		0.180		0.195		1.000		0.076		0.480		0.382		0.083	
	*P* value	<.001		<.001		<.001		<.001		<.001		—		<.001		<.001		<.001		<.001	
**HL**																					
	*r*	0.202		0.038		0.123		0.230		0.092		0.076		1.000		0.096		0.093		0.009	
	*P* value	<.001		<.001		<.001		<.001		<.001		<.001		—		<.001		<.001		.24	
**LWORD**																					
	*r*	0.353		0.147		0.330		0.258		0.254		0.480		0.096		1.000		0.398		0.107	
	*P* value	<.001		<.001		<.001		<.001		<.001		<.001		<.001		—		<.001		<.001	
**LTENURE**																					
	*r*	0.286		0.166		0.320		0.134		0.350		0.382		0.093		0.398		1.000		0.109	
	*P* value	<.001		<.001		<.001		<.001		<.001		<.001		<.001		<.001		—		<.001	
**LCT**																					
	*r*	0.390		0.151		0.138		0.100		0.252		0.083		0.009		0.107		0.109		1.000	
	*P* value	<.001		<.001		<.001		<.001		<.001		<.001		.24		<.001		<.001		—	

^a^LPRICE: natural logarithm of consulting price.

^b^LWAGE: natural logarithm of provincial wage level.

^c^LWVOL: natural logarithm of the doctor’s electronic word of mouth volume.

^d^WVAL: doctor’s electronic word of mouth valence.

^e^LPSALE: natural logarithm of past online sales.

^f^CL: clinic title.

^g^HL: hospital level.

^h^LWORD: natural logarithm of the length of the doctor’s profile.

^i^LTENURE: natural logarithm of the tenure with Haodf.

^j^LCT: length of consulting time.

^k^Not applicable.

**Table 4:** Results of two-sample t tests.

Variable		Mean (SD)	*t* value	*P* value
**Clinic title (CL)**			43.395	<.001
	Low	49.324 (52.038)		
	High	98.269 (87.516)		
**Hospital level (HL)**			17.618	<.001
	Low	53.666 (72.607)		
	High	88.088 (82.542)		
**Doctor’s eWOM^a^ volume (WVOL)**			40.670	<.001
	Low	59.552 (63.915)		
	High	109.922 (90.475)		
**Doctor’s eWOM valence (WVAL)**			34.004	<.001
	Low	66.630 (69.581)		
	High	112.881 (92.010)		
**Past online sales (PSALE)**			46.096	<.001
	Low	57.502 (57.557)		
	High	115.244 (94.243)		
**Provincial wage level (WAGE)**			38.687	<.001
	Low	62.776 (60.232)		
	High	114.622 (97.464)		

^a^eWOM: electronic word of mouth.

### HLM Analysis

#### Null Model

We hypothesized that both individual- and province-level variables would be significantly correlated with consulting fees. For these hypotheses to be supported, there had to be significant between-province variance in consulting fees. Therefore, we used HLM to estimate a null model as in Eq (1), in which no independent variables are specified for either the level-1 or level-2 function, to test the significance level of the between-groups variance in the dependent variable by examining the significance level of 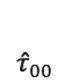 and the ICC. In our case, 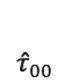
=0*.*108 (*P<.*001); thus, we rejected the null hypothesis (*τ*_00_=0), which implies a significant difference of LPRICE between different provinces. In addition, the ICC was calculated to be 0.142 according to Eq (2), which means that there is a 14.2% difference in the consulting fee between provinces, whereas there is an 85.8% difference within provinces. According to the criteria recommended by James [62] and Cohen [63], the impact of between-groups variance should not be ignored in the context of our study. In other words, our data met the prerequisites for performing HLM analysis rather than the traditional regression models. Therefore, we proceeded to test our hypotheses using HLM.

As suggested by Hofmann and Gavin [64], for each estimated HLM model (described below), all independent variables at level 1 were grand mean–centered. In addition, we defined *R^2^_within-group_* as the proportion of within-group variance explained by the specific model compared with the null model and *R^2^_between-groups_* as the proportion of between-group variance explained by the specific model compared with the null model. Moreover, following the formula reported in Raudenbush and Bryk [26], we further calculated the total variance explained by the dependent variable as follows:

R^2^_total_=R^2^_within-group_×(1 – ICC)+R^2^_between-groups_×ICC **(5)**

where ICC is defined as in Eq (2).

#### Random Intercept Model

Table 5 presents the HLM analysis results, including the coefficients, standard errors, *t* values, and *P* values, for the two models with the sample of 16,008 physicians. In the random intercept model as in Eq (3), the results showed a positive relationship between doctors’ wage levels and online health care consulting price, thereby supporting H1 (*R^2^_within-group_*=0.431, *R^2^_between-groups_*=0.769, *R^2^_total_*=0.479). Thus, when a doctor comes from a province with a higher wage, they generally charge a higher price for online health care consulting. We also found that the online reputation of doctors indicated by variables such as eWOM volume, eWOM valence, and past sales had positive associations with the online health care consulting price, as represented by the corresponding coefficients, which in turn supports H2a, H2b, and H2c. In other words, when a doctor receives more WOM volume, has a higher eWOM rating, and has had more sales in the past, they charge a higher consulting fee. Finally, a significant positive relationship between offline reputation (such as clinic title and hospital level) and consulting price was found, which supports H3a and H3b, suggesting that consulting price results from higher clinic titles or a higher-level hospital. All the estimates were statistically significant (*P<.*001).

**Table 5:** Hierarchical linear model results for the effects of determinants on online health care consulting prices.

Variable			Random intercept model					Random coefficient model			
			Coefficient^a^ (SE)	*t* value^b^		*P* value	Coefficient^a^		*t* value		*P* value
Level-2 variable: LWAGE^c^			0.751 (0.149)	5.050		<.001	0.730 (0.067)		10.893		<.001
**Level-1 variables**											
	Intercept	−0.537 (0.048)		–11.203	<.001		−0.619 (0.036)		–17.230	<.001	
	LWVOL^d^	0.060 (0.006)		9.876	<.001		0.069 (0.012)		5.598	<.001	
	WVAL^e^	0.412 (0.022)		18.551	<.001		0.371 (0.037)		10.027	<.001	
	LPSALE^f^	0.091 (0.004)		20.566	<.001		0.073 (0.008)		8.805	<.001	
	CL^g^	0.378 (0.013)		29.304	<.001		0.411 (0.017)		24.345	<.001	
	HL^h^	0.325 (0.017)		19.207	<.001		0.320 (0.017)		18.996	<.001	
**Control Variables**											
	LWORD^i^	0.079 (0.006)		12.523	<.001		0.071 (0.006)		11.056	<.001	
	LTENURE^j^	0.017 (0.008)		2.275	.02		0.013 (0.008)		1.766	.08	
	LCT^k^	0.082 (0.002)		45.378	<.001		0.082 (0.002)		45.624	<.001	
	DIV^l^: Surgery	−0.128 (0.017)		–7.715	<.001		−0.131 (0.017)		–7.921	<.001	
	DIV: Gynecology-obstetrics	0.059 (0.021)		2.745	.006		0.062 (0.021)		2.881	.004	
	DIV: Pediatrics	0.049 (0.020)		2.490	.01		0.055 (0.020)		2.778	.06	
	DIV: Orthopedics	−0.211 (0.023)		–9.121	<.001		−0.212 (0.023)		–9.187	<.001	
	DIV: Ophthalmology	−0.018 (0.026)		–0.694	.49		−0.017 (0.026)		–0.651	.52	
	DIV: Oral health	−0.189 (0.031)		–6.021	<.001		−0.191 (0.031)		–6.118	<.001	
	DIV: Cancer	−0.047 (0.030)		–1.585	.11		−0.042 (0.029)		–1.424	.15	
	DIV: Chinese medicine	−0.345 (0.030)		–16.375	<.001		−0.337 (0.021)		–16.023	<.001	
	DIV: Others	0.024 (0.017)		1.394	.16		0.026 (0.017)		1.510	.13	

^a^Standardized regression coefficient.

^b^Degrees of freedom=28 for provincial wage level and 15,961 for the other variables.

^c^LWAGE: natural logarithm of provincial wage level.

^d^LWVOL: natural logarithm of doctor’s electronic word of mouth volume.

^e^WVAL: doctor’s electronic word of mouth valence.

^f^LPSALE: natural logarithm of past online sales.

^g^CL: clinic title.

^h^HL: hospital level.

^i^LWORD: natural logarithm of the length of the doctor’s profile.

^j^LTENURE: natural logarithm of the doctor’s tenure on Haodf.

^k^LCT: length of consulting time.

^l^DIV: division.

#### Random Coefficient Model

Even when adopting the random coefficient model, which allows the coefficients in all groups to be different, the main characteristics of these results also showed positive associations with the online health care consulting price (Table 5; *R^2^_within-groups_*=0.437, *R^2^_between-groups_*=0.730). In comparison with the null model, the *R^2^_total_* of the random intercept and random coefficient models were both exactly 0.479, implying that both models represent an obvious improvement over the null model and better explain the online health care consulting price. Additionally, the effects of control variables, LWORD and LCT, were significantly positive and were all consistent in both models. However, the effect of LTENURE was significantly positive only in the random intercept model. In summary, based on the above outcomes, all of the proposed hypotheses were clearly supported. We will provide further evidence supporting the robustness of our results in the next subsection.

### Robustness Tests for the Subsample

To test for robustness, a subsample was selected based on two different criteria. First, it is necessary to certify the authenticity of the observed online health care consulting prices. As mentioned above, the sample included only doctors who had completed at least one consultation at their own requested price. Nevertheless, it might be argued that the number of transactions is too small, which would make the online health care consulting price not suitable as a reference point. Thus, we verified whether the HLM results were robust for doctors with at least 10 past sales. Specifically, we ignored relatively inactive physicians, resulting in a reduction of the sample size to 4055. Regarding this subsample, since the random intercept and random coefficient models produced similar results in all robustness tests, we show only the results of the random coefficient model in Multimedia Appendix 1. The coefficients were substantially similar to those presented in Table 5. Second, we further examined the robustness of different subperiods. Specifically, we divided all of the doctors into two subsamples based on whether they had joined the Haodf website before or after January 1, 2014. We chose this date as the department sample for two main reasons. First, this point was chosen to better understand whether the length of time as a member on the platform would produce consistent results for our proposed model. Second, since the Haodf platform provided more complete mobile apps after 2014, we believe that doctors who joined the platform after this date may be more inclined to use a phone to communicate with their patients. Two subsamples were used: Subsample 2 and Subsample 3. Multimedia Appendix 1 shows that all independent variables still had significantly positive values. In summary, the additional empirical evidence provided here further confirmed the robustness of the relationship between the level-2 and level-1 variables and online health care consulting prices.

## Discussion

### Principal Results

The development of the OHC has become increasingly important, especially in countries where there are relatively few medical resources such as China [7,8]. A common type of service in the OHC is online health care consultation, which allows patients and doctors to make contact and exchange medical information remotely [59,65]. Since reasonable and stable prices are an important factor in ensuring the sustainable development of any industry, we have focused on investigating the determinants of online health care consulting prices. To the best of our knowledge, this study is the first to target this topic through analysis of large-scale empirical data. The main theoretical contribution of this study is that of enhancing and complementing the past service pricing literature to make it more applicable to the OHC. Specifically, the consideration of both cost-based and competition-based pricing approaches involves service characteristics [31,33]; however, there are many differences between the features of online health care consultation and general services, such as more specialized service providers, zero fixed costs, and online service in online health care consultation. Thus, we propose six determinants of online health care consulting prices to bridge this gap in knowledge of this field. Our empirical results support the conclusion that working area wage levels, eWOM volume, eWOM valence, past sales, the doctor’s clinic title, and the level of the doctor’s affiliated hospital have significantly positive effects on the doctor’s online health care consulting price. Moreover, we suggest some corresponding management implications.

From the patients’ perspective, since doctors might exploit informational asymmetry to defraud them [17], patients hope to avoid being subjected to an overcharge or low-quality service. We suggest that in practice, patients can first determine a basic price that is calculated based on the average price of other doctors with a similar specialty and then further adjust their expected price through our proposed determinants. For instance, when the price requested by the doctor is higher than the basic price, patients can check if this doctor is a chief physician, the eWOM is better, the number of past sales is larger, or the doctor is from a big city. From the opposite perspective, doctors can set reasonable or acceptable prices after reviewing their personal conditions based on certain proposed determinants. In particular, similar to the results in previous studies, our results also verified that eWOM valence and volume have positive effects on prices [43,44]. Therefore, doctors who want to offer online health care consulting services should manage their online reputation well. Companies providing online health care services, such as the Haodf website, should construct an effective feedback machine that can increase the patient’s trust or reduce the uncertainty of online transactions [23]. Eliminating information asymmetry is definitely one of the pressing tasks in developing the OHC industry. Finally, our findings may be of interest to government authorities who wish to develop the OHC. There is no doubt that online health care services can indeed break through geographical limits and give rural residents the opportunity to obtain medical service from big cities. However, doctors from cities often charge higher fees because of the higher opportunity cost. One of the most important functions of the OHC is to reduce the medical disparity between urban and rural areas; however, planners seem to ignore the fact that rural residents with lower incomes may not be able to afford such high medical consultation costs. Therefore, the government can provide incentives to encourage urban doctors to give some discounts to rural residents or can directly provide appropriate subsidies. We believe that specific development of the OHC should not be undertaken simply for the sake of convenience. More importantly, it should be the case that people who truly need medical resources can receive effective medical care immediately.

### Limitations and Future Work

There are some limitations to this study as well as indications of possible directions for future work. First, a cross-sectional study design was used for data collection in this study. The sample might have led to results that are time-sensitive. For example, diseases prevalent in different seasons can affect the behavior of physicians. In addition, the Haodf platform regularly provides discounted prices to a certain number of doctors. Cross-sectional data may limit the range of explanations for price changes; future studies should address this limitation by extending the panel data to accurately simulate price changes over time while reducing time-sensitivity issues. Second, all of the empirical data were collected from the Haodf website. Although the choice in this study to use only one OHC helped us to improve internal validity, it can also reduce the generalizability of our findings, and future research should validate our results in other OHCs. In particular, samples of remote medical services provided by traditional hospitals can be included. It is worth studying whether the provision of online health care services by traditional hospitals or through internet companies can bring more benefits to society as a whole. Finally, due to the availability of data and to ethical considerations, we did not include any patient-related information but instead used only the doctors’ public information. In future studies, under the condition of having obtained patients’ privacy permission, researchers could discuss the factors affecting online health care consulting prices from the perspective of patient needs.

### Conclusions

This study employed data collected from the Haodf website, which includes information related to 16,008 doctors, 1682 different hospitals, and 30 provinces in China. The main findings can be summarized as follows. Our empirical results support the conclusion that five level-1 variables (the doctor’s eWOM volume, eWOM valence, past sales, clinic title, and hospital level) have significantly positive effects on the online health care consulting price. In other words, the doctor’s online and offline reputation are both meaningful signals that affect their consulting price. In addition, the level-2 variable (provincial wage level) also had a significantly positive impact on the consulting price. The beauty of the internet is that it breaks through geographical restrictions and ideally makes it possible for anyone to access online services due to the relatively low threshold of information transmission. Nevertheless, the wage level in the geographical area in which the doctor works affects the charges for different online consulting services. This suggests that the internet can indeed reduce the cost of delivering information, but it might not eliminate the barriers leading to differences in urban and rural consumption levels.

## Appendix

Multimedia Appendix 1 Robustness tests for the determinants of online health care consulting prices.

## Abbreviations

eWOM: electronic word of mouth
HLM: hierarchical linear modeling
ICC: intraclass correlation coefficient
OHC: online healthcare community
