# Supplementary material for: Consultation Pricing of the Online Health Care Service in China: Hierarchical Linear Regression Approach
Source: J Med Internet Res. 2021 Jul 14;23(7):e29170. doi: 10.2196/29170 (PMC8319787; doi:10.2196/29170)
Supplement: Multimedia Appendix 1 [file jmir_v23i7e29170_app1.docx]

**Multimedia Appendix 1.** Robustness tests for the determinants of online health care consulting prices.

| Variables | | Subsample 1 (n=4055 observations) | | | | Subsample 2 (n=9982 observations) | | | | Subsample 3 (n=6026 observations) | | | |
| --- | --- | --- | --- | --- | --- | --- | --- | --- | --- | --- | --- | --- | --- |
|  | | Coefficient^a^ | SE | *t* value^b^ | *P* value | Coefficient | SE | *t* value^c^ | *P* value | Coefficient | SE | *t* value^d^ | *P* value |
|  | |  |  |  |  |  |  |  |  |  |  |  |  |
| Level-2 variable: LWAGE^e^ | | 0.669 | 0.106 | 6.285 | <.001 | 0.831 | 0.092 | 8.992 | <.001 | 0.591 | 0.071 | 8.338 | <.001 |
| **Level-1 Variables** | |  |  |  |  |  |  |  |  |  |  |  |  |
|  | Intercept | −0.648 | 0.064 | –10.158 | <.001 | −0.581 | 0.040 | –14.468 | <.001 | −0.446 | 0.058 | –7.744 | <.001 |
|  | LWVOL^f^ | 0.097 | 0.019 | 4.983 | <.001 | 0.043 | 0.012 | 3.493 | <.001 | 0.105 | 0.016 | 6.580 | <.001 |
|  | WVAL^g^ | 0.140 | 0.039 | 3.564 | <.001 | 0.441 | 0.039 | 11.311 | <.001 | 0.327 | 0.049 | 6.729 | <.001 |
|  | LPSALE^h^ | 0.085 | 0.013 | 6.269 | <.001 | 0.065 | 0.009 | 7.164 | <.001 | 0.082 | 0.012 | 6.635 | <.001 |
|  | CL^i^ | 0.438 | 0.037 | 11.745 | <.001 | 0.383 | 0.020 | 18.942 | <.001 | 0.313 | 0.039 | 7.933 | <.001 |
|  | HL^j^ | 0.319 | 0.039 | 8.435 | <.001 | 0.351 | 0.021 | 17.042 | <.001 | 0.240 | 0.030 | 8.114 | <.001 |
| **Control Variables** | |  |  |  |  |  |  |  |  |  |  |  |  |
|  | LWORD^k^ | 0.059 | 0.011 | 5.197 | <.001 | 0.085 | 0.008 | 10.294 | <.001 | 0.050 | 0.010 | 4.870 | <.001 |
|  | LTENURE^l^ | 0.071 | 0.021 | 3.445 | <.001 | 0.022 | 0.010 | 2.262 | .02 | 0.123 | 0.068 | 1.807 | .07 |
|  | LCT^m^ | 0.062 | 0.003 | 21.803 | <.001 | 0.082 | 0.002 | 34.796 | <.001 | 0.082 | 0.003 | 29.152 | <.001 |
|  | DIV^n^: Surgery | −0.174 | 0.032 | –5.465 | <.001 | −0.130 | 0.021 | –6.194 | <.001 | −0.134 | 0.027 | –4.914 | <.001 |
|  | DIV: Gynecology-obstetrics | 0.031 | 0.037 | 0.857 | .39 | 0.068 | 0.026 | 2.652 | .008 | 0.063 | 0.039 | 1.633 | .10 |
|  | DIV: Pediatrics | –0.066 | 0.034 | –1.963 | .05 | 0.100 | 0.023 | 4.289 | <.001 | -0.050 | 0.037 | –1.363 | .17 |
|  | DIV: Orthopedics | −0.258 | 0.047 | –5.454 | <.001 | −0.194 | 0.030 | –6.486 | <.001 | −0.236 | 0.037 | –6.450 | <.001 |
|  | DIV: Ophthalmology | −0.056 | 0.046 | –1.207 | .23 | −0.012 | 0.034 | –0.354 | .72 | −0.043 | 0.042 | –1.020 | .31 |
|  | DIV: Oral health | −0.268 | 0.063 | –4.240 | <.001 | −0.191 | 0.039 | –4.957 | <.001 | −0.199 | 0.053 | –3.752 | <.001 |
|  | DIV: Cancer | 0.006 | 0.051 | 0.109 | .91 | −0.061 | 0.038 | –1.579 | .11 | −0.026 | 0.046 | –0.572 | .57 |
|  | DIV: Chinese medicine | −0.329 | 0.037 | –8.903 | <.001 | −0.353 | 0.029 | –12.252 | <.001 | −0.332 | 0.032 | –10.422 | <.001 |
|  | DIV: Others | –0.069 | 0.031 | –2.245 | .03 | 0.050 | 0.021 | 2.337 | .02 | -0.016 | 0.029 | –0.552 | .58 |
| *R*^2^within-group | | 0.330 | —^o^ | — | — | 0.419 | — | — | — | 0.421 | — | — | — |
| *R*^2^between-group | | 0.597 | — | — | — | 0.721 | — | — | — | 0.740 | — | — | — |
| *R*^2^total^p^ | | 0.375 | — | — | — | 0.461 | — | — | — | 0.457 | — | — | — |

^a^Standardized regression coefficient.

^b^Degrees of freedom=28 for provincial wage levels and 4008 for all other variables.

^c^Degrees of freedom=28 for provincial wage levels and 9935 for all other variables.

^d^Degrees of freedom=28 for provincial wage levels and 5979 for all other variables.

^e^LWAGE: natural logarithm of provincial wage level.

^f^LWVOL: natural logarithm of doctor’s electronic word of mouth volume.

^g^WVAL: doctor’s electronic word of mouth valence.

^h^LPSALE: natural logarithm of past online sales.

^i^CL: clinic title.

^j^HL: hospital level.

^k^LWORD: natural logarithm of the length of the doctor’s profile.

^l^LTENURE: natural logarithm of the doctor’s tenure on Haodf.

^m^LCT: length of consulting time.

^n^DIV: division.

^o^Not applicable.

^p^*R*^2^total=*R*^2^_within-group_ ×(1 − ICC)+*R*^2^_between-group_ × ICC, where ICC represents the ratio of the between-group variance to the total variance based on the corresponding null model. For these three subsamples, the values of ICC are 0.167, 0.137, and 0.113, respectively.
